# A 2012 Workshop: Vaccine and Drug Ontology in the Study of Mechanism and Effect (VDOSME 2012)

**DOI:** 10.1186/2041-1480-3-12

**Published:** 2012-12-18

**Authors:** Yongqun He, Luca Toldo, Gully Burns, Cui Tao, Darrell R Abernethy

**Affiliations:** 1 Unit for Laboratory Animal Medicine, Department of Microbiology and Immunology, and Center for Computational Medicine and Bioinformatics, University of Michigan Medical School, Ann Arbor, MI, USA; 2 Merck KGaA, Frankfurter Straße 250, Darmstadt, 64293, Germany; 3 Information Sciences Institute, 4676 Admiralty Way, Suite 1001, Marina del Rey, CA, 90292, USA; 4 Division of Biomedical Statistics and Informatics, Department of Health Sciences Research, Mayo Clinic, Rochester, MN, USA; 5 U.S. Food and Drug Administration (FDA), Johns Hopkins School of Medicine, Baltimore, MD, USA

## Abstract

Vaccines and drugs have contributed to dramatic improvements in public health worldwide. Over the last decade, there have been efforts in developing biomedical ontologies that represent various areas associated with vaccines and drugs. These ontologies combined with existing health and clinical terminology systems (*e.g.*, SNOMED, RxNorm, NDF-RT, MedDRA, VO, OAE, and AERO) could play significant roles on clinical and translational research. The first “Vaccine and Drug Ontology in the Study of Mechanism and Effect” workshop (VDOSME 2012) provided a platform for discussing problems and solutions in the development and application of biomedical ontologies in representing and analyzing vaccines/drugs, vaccine/drug administrations, vaccine/drug-induced immune responses (including positive host responses and adverse events), and similar topics. The workshop covered two main areas: (i) ontologies of vaccines, of drugs, and of studies thereof; and (ii) analysis of administration, mechanism and effect in terms of representations based on such ontologies. Six full-length papers included in this thematic issue focus on ontology representation and time analysis of vaccine/drug administration and host responses (including positive immune responses and adverse events), vaccine and drug adverse event text mining, and ontology-based Semantic Web applications. The workshop, together with the follow-up activities and personal meetings, provided a wonderful platform for the researchers and scientists in the vaccine and drug communities to demonstrate research progresses, share ideas, address questions, and promote collaborations for better representation and analysis of vaccine and drug-related terminologies and clinical and research data.

## Introduction and background

Innovative therapeutic interventions are critical to prevent and treat human and animal diseases. A way to broadly classify therapeutic interventions is through their timing in administration: Vaccines are classically administered to prevent the appearance of a medical problem, while drugs are generally administered to treat a medical problem. Noticeable exceptions can be found for both classes of therapeutic interventions such as cancer vaccines (that are administered after detection of the problem), and protein pump inhibitors (that are often administered to prevent gastric problems in co-therapy with other drugs or in specific hospital settings). Nevertheless, vaccines and drugs are similarly regulated both in research and development, manufacturing, clinical trials, government approval and regulation, and post-licensing usage surveillance and monitoring. In a broader scope, vaccine is a special type of drug. Vaccines and drugs also have many differences. For example, for vaccines, dose, time, route, and frequency of administration are generally known quite precisely. However, since drugs are used for patients with different conditions, dose, time, and frequency of drug administration are often very difficult to establish. Since vaccines are often administered to healthy people to prevent medical problems, attribution of an adverse event following vaccination is less likely to be confounded by signs or symptoms of underlying medical problems as it is with drugs that are administered to treat medical problems. However, separation of manifestation of medical problem from manifestation of drug adverse event is often very challenging. In the U.S.A, vaccines are regulated under different laws by the Center for Biologics (CBER) at FDA, while drugs are regulated under the Food Drug and Cosmetic Act by the Center for Drugs (CDER) at FDA. Safety surveillance for vaccines is for the most part carried out by the Center for Disease Control (CDC) in Atlanta, while for drugs it is carried out by the FDA. Due to these similarities and differences between vaccines and drugs, a closer communication between these two areas is important to create effective ontological frameworks around which we can build comparative and predictive systems for both vaccines and drugs.

Although several related ontologies have been initiated with much progress made in the recent years, we are still facing many challenges for fully and logically representing vaccines and drugs and efficiently leveraging the ontologies. In the case of ontology representation, no consensuses have been achieved on how to ontologically represent many relevant areas, for example: (i) vaccine and drug administration dose, route, and frequency, (ii) drug-drug interactions, drug-food interactions, and how they affect vaccine and drug adverse events, (iii) experimental testing and analysis of vaccine/drug-induced immune responses, and (iv) the complexity of time constraints for clinical events post vaccination or medication. Meanwhile, it is also a challenge to efficiently apply biomedical ontologies to solve research and clinical problems. For example, is that possible to apply ontologies for advanced literature mining in order to discover new gene interaction networks underlying protective immunity or vaccine and drug adverse events? How to apply ontologies for personalized medicine? How to use ontologies to improve the performance of complex vaccine/drug research and clinical data analysis?

The full-day “Vaccine and Drug Ontology in the Study of Mechanism and Effect” workshop (VDOSME 2012) aspires to become an international forum for researchers to identify, propose, and discuss solutions for important research problems in ontology representation and analysis of vaccine and drug formation and preparation, administration, function mechanisms, and induced host immune responses. The immune responses can be positive responses for prevention and/or treatment of a disease, or can be negative responses, i.e., adverse events. This workshop aimed to support the deeper understanding of vaccine and drug mechanisms and effects.

VDOSME 2012 was held on July 21, 2012, at Graz, Germany. This workshop was part of the third International Conference on Biomedical Ontology (ICBO 2012). Detailed information about VDOSME 2012 is available on the web: http://kr-med.org/icbofois2012/vdosme/. Fourteen people attended the workshop. The attendees included paper presenters, senior academic and government scientists (e.g., Dr. Laszlo Balkanyi, knowledge manager, European Centre for Disease Prevention and Control or ECDC), postdoctoral fellows, and graduate students. There were nine submissions in total. After peer reviewing of each paper by at least three independent reviewers, six full-length papers and one short-length papers were accepted for proceeding paper publications and oral presentations in the workshop. One of the six papers came from the transfer from another ICBO 2012 workshop ‘‘Methods for adverse events representation Ontology and Information Model’’. Dr. Cui Tao, one oral presenter and co-organizer, presented her talk from the USA through a teleconference system. After two additional rounds of independent peer reviewing by the workshop co-organizers and the journal editors, the selected six full-length papers were extended and accepted for publication in the current issue of the Journal of Biomedical Semantics (JBMS).

## Summary of selected papers in the thematic issue

The six papers selected for this thematic issue are extended versions of the original full-length papers presented at the VDOSME 2012 [1-6]. These papers cover a wide range of topics including ontology modeling and analysis, ontology-based knowledge extraction from big data case reports and literature, and ontology-based Semantic Web applications.

In the area of ontology modeling and representation, Lin and He introduce the ontological classification and definitions of different vaccine components and vaccine administrations within the framework of the Vaccine Ontology (VO) [1]. Their studies show that vaccine formulation and administration route can independently or collaboratively affect the outcomes of vaccine-induced host responses. Tao et al. introduce a novel method for using ontologies (including Time Event Ontology or TEO, VO, and Ontology of Adverse Events or OAE) and semantic web technologies to represent and reason temporal information and relations for post-vaccination events reported in the US FDA/CDC Vaccine Adverse Event Reporting System (VAERS) [2]. Effective analyses of time trends for post-vaccine adverse events (AEs) can enhance clinical research in different areas such as vaccine safety analyses, causality assessments, and retrospective studies.

In the area of ontology-based text mining, Gurulingappa et al. outline an adaption of a machine learning-based system for the identification and extraction of the relations between drugs and adverse effects from MEDLINE case reports [3]. The authors used an ontology-driven approach for the manual creation of the corpus used for benchmarking, mapped the annotation ontology with the Ontology of Adverse Events (OAE), and share a vision on OAE-driven information extraction. Using all PubMed abstracts and the information in the VO, Hur et al. applied a genome-wide, centrality and ontology-based literature mining approach to study fever and vaccination-associated gene interaction networks [4]. This study identified a generic fever network consisting more than 400 genes and a subset of the network (29 genes) associated with vaccines. Detailed analysis of these gene networks allows new scientific discoveries and hypothesis generations. After the publication of this work, Botsis et al. reported another vaccine adverse event text mining (VaeTM) system based on a semantic text mining strategy, which has a sensitivity of 83.1% to detect symptoms, diagnoses, drugs, vaccines, and anatomical terms [7]. These works show how much the vaccine community is leveraging upon text mining.

In ontology-based Semantic Web applications, Zhu et al. present a Semantic Web technology framework to map the Structured Product Labeling (SPL) drug labels with existing drug ontologies: NDF-RT and RxNorm [5]. The profiling outcomes support meaningful use of SPL drug labels in clinical applications through standard NDF-RT and RxNorm. In another paper, Doulaverakis et al. present a semantic enabled online service called GalenOWL that offers real time drug-drug and drug-disease interaction discovery [6]. A rule based inference method combining ontology information was developed to provide drug recommendations. These OWL-based Semantic Web applications provide excellent use case studies on translating ontology research into possibly very useful medical tool development.

## Workshop presentations and discussions

In the workshop, the six full-length papers described above were orally presented. In addition, Dr. Gully Burns also presented his short-paper on the development and usage of “Ontology of Experimental Variables and Values” (OoEVV) for interlinking multiple experimental variables that measure the same underlying quality but are expressed using different measurement scales [8]. Furthermore, three general discussion sessions were arranged and moderated by our workshop co-organizers. Dr. Luca Toldo moderated a discussion session on the topic "Relation between drug and vaccine ontology informatics". In this session, we discussed: (i) Similarities and differences between drug and vaccine informatics, (ii) Collaborations between drug and vaccine ontology informatics fields, and (iii) Collaborations between academia and industry. The second discussion session was on the updates of the development of adverse event-related ontologies. Melanie Courtot was invited to update the development of the Adverse Event Reporting Ontology (AERO), and Dr. Yongqun He updated the development of the OAE and its applications. The relation and coordination between AERO and OAE were discussed. Dr. Gully Burns moderated the discussion session "Challenges and opportunities". We discussed current achievements and challenges and international community collaborations. The workshop attendees all agreed that such a workshop was very beneficial to the community-based vaccine and drug-related ontology development and applications. All attendees would like to welcome more VDOSME or similar workshops in the coming years. The VDOSME workshop, together with many activities derived from the workshop, has provided an effective platform for the researchers in the vaccine and drug communities to share research progresses and ideas, discuss problems and solutions, and promote interdisciplinary collaborations.

## Competing interests

The authors declare that they have no competing interests.
